# Cooler canopy leverages sorghum adaptation to drought and heat stress

**DOI:** 10.1038/s41598-022-08590-6

**Published:** 2022-03-17

**Authors:** Aliza Pradhan, Lalitkumar Aher, Vinay Hegde, Krishna Kumar Jangid, Jagadish Rane

**Affiliations:** 1 ICAR-National Institute of Abiotic Stress Management, Malegaon, Baramati, Pune, MH 413115 India; 2 Dr. Panjabrao Deshmukh Krishi Vidyapeeth, Akola, MH 444104 India

**Keywords:** Plant physiology, Plant stress responses

## Abstract

In the present study, individual and combined effects of drought and heat stress were investigated on key physiological parameters (canopy temperature, membrane stability index, chlorophyll content, relative water content, and chlorophyll fluorescence) in two popular sorghum cultivars (*Sorghum bicolor* cvs. Phule Revati and Phule Vasudha) during the seedling stage. Estimating canopy temperature through pixel-wise analysis of thermal images of plants differentiated the stress responses of sorghum cultivars more effectively than the conventional way of recording canopy temperature. Cultivar difference in maintaining the canopy temperature was also responsible for much of the variation found in critical plant physiological parameters such as cell membrane stability, chlorophyll content, and chlorophyll fluorescence in plants exposed to stress. Hence, the combined stress of drought and heat was more adverse than their individual impacts. The continued loss of water coupled with high-temperature exposure exacerbated the adverse effect of stresses with a remarkable increase in canopy temperature. However, Phule Vasudha, being a drought-tolerant variety, was relatively less affected by the imposed stress conditions than Phule Revati. Besides, the methodology of measuring and reporting plant canopy temperature, which emerged from this study, can effectively differentiate the sorghum genotypes under the combined stress of drought and heat. It can help select promising genotypes among the breeding lines and integrating the concept in the protocol for precision water management in crops like sorghum.

## Introduction

Climate change is projected to raise the occurrence and distribution of abiotic stresses, posing a severe threat to crop production and food security^1,2^. Abiotic stresses, in particular, drought and heat, are the significant factors limiting the crop yield in arid and semi-arid regions, where sorghum (*Sorghum bicolor* (L.) Moench) is an essential component of the cropping systems^3^. It is considered the fifth major cereal crop and holds significant global importance for its use as food, feed, industry raw material and bio-energy production^4^. However, most of the sorghum producing regions often experience high temperature (heat stress) combined with irregular water availability (drought stress), and the events are likely to be higher in terms of their frequency and severity than previously anticipated^5,6^. Under natural crop growing conditions, concurrent occurrence of moisture deficit and high temperature can adversely affect plant growth, development and yield performance, especially in tropical and sub-tropical climates^7–10^. Therefore, it is crucial that both drought and heat stress should be considered simultaneously as their combined impact is different than their individual occurrences^11,12^.

It is well documented that the effects of drought, heat, and their combination varies among different crops as well as different genotypes within a specific crop/plant species^12,13^. The combined stress due to drought and heat had a significantly higher damaging impact than each of its individual stress components in wheat, sorghum, barley, and maize^14–18^. Much of the impact due to stress results from an effect on crucial physiological processes such as canopy temperature, membrane stability, chlorophyll content, tissue water retention, and photosynthesis as they are directly involved in maintaining the photosynthetic potential and primary production of assimilates^12,14,19^. In general, change in canopy temperature is one of the primary and rapid physiological responses when plants are subjected to high temperature and drought, as reported in wheat^20^. Since transpiration plays a key role in leaf water status and leaf temperature variations, canopy temperature has been established as an indicator to represent the capability of the plants to balance tissue moisture loss and protection from overheating^20,22^. Heat stress results in higher stomatal conductance as the plant tries to maintain a cooler canopy by transpiration, while drought has the opposite impact of preventing moisture loss^23^. The photosynthesis gets affected by elevated leaf temperature in response to high ambient temperature only or in combination with drought due to reduced stomatal conductance as in cotton^24^ and *Nicotiana tabacum*^25^. Stress-induced decrease in photosynthesis has also been attributed to either Rubisco destabilization or PS II damage^26^. Enhanced Rubisco activity was reported in response to heat stress, whereas it reduced in response to drought and their combined stress condition^27^. These results suggest that maintenance of enabling canopy temperature for optimum photosynthetic efficiency is vital for crop acclimation to a combination of drought and heat stress.

Further, the stress sensitivity of plants is determined by their developmental stages. Early-season drought coupled with elevated temperature can cause impaired seedling growth and establishment^28,29^. Currently, there is a lack of information on the effects of drought, heat, and their interaction during the seedling establishment stage, particularly in cultivars released for the arid and semi-arid region. An insight into these aspects can provide a benchmark for further improvement in the abiotic stress tolerance of sorghum.

Hence, the present study was mainly focused on understanding the independent and combined effects of drought and heat stress on canopy temperature and its association with other vital physiological parameters of two popular sorghum cultivars (Phule Vasudha and Phule Revati) during the seedling stage. The study aimed to test the hypothesis that cooler canopy is more critical when sorghum plants are exposed to both drought stress and high temperature than when any one of them occur during seedling stage. Hence, the key objective of this research was to assess cultivar variation in maintaining the canopy temperature under elevated ambient temperature with or without soil moisture depletion and its relation with vital physiological processes such as cell membrane stability, chlorophyll content, tissue water retention, and PS-II activity.

## Results

The present study aimed at the individual and combined effects of drought and heat stress on critical physiological traits. It was primarily focused on canopy temperature dynamics, cell membrane stability, chlorophyll content, tissue water content, and sensitivity of PS-II. Though the data were recorded on the third, fifth and seventh days after the imposition of stress treatments, and there was no significant effect of treatments on day three and day five (data not reported); hence the data of day seven were analyzed and reported. This section has been organized to explain the hypothesis that variation in plant canopy temperature can explain the effects of differential responses to drought, heat, and their combination in sorghum cultivars.

### Comparative response of canopy temperature to stress treatments

**Figure 1:**
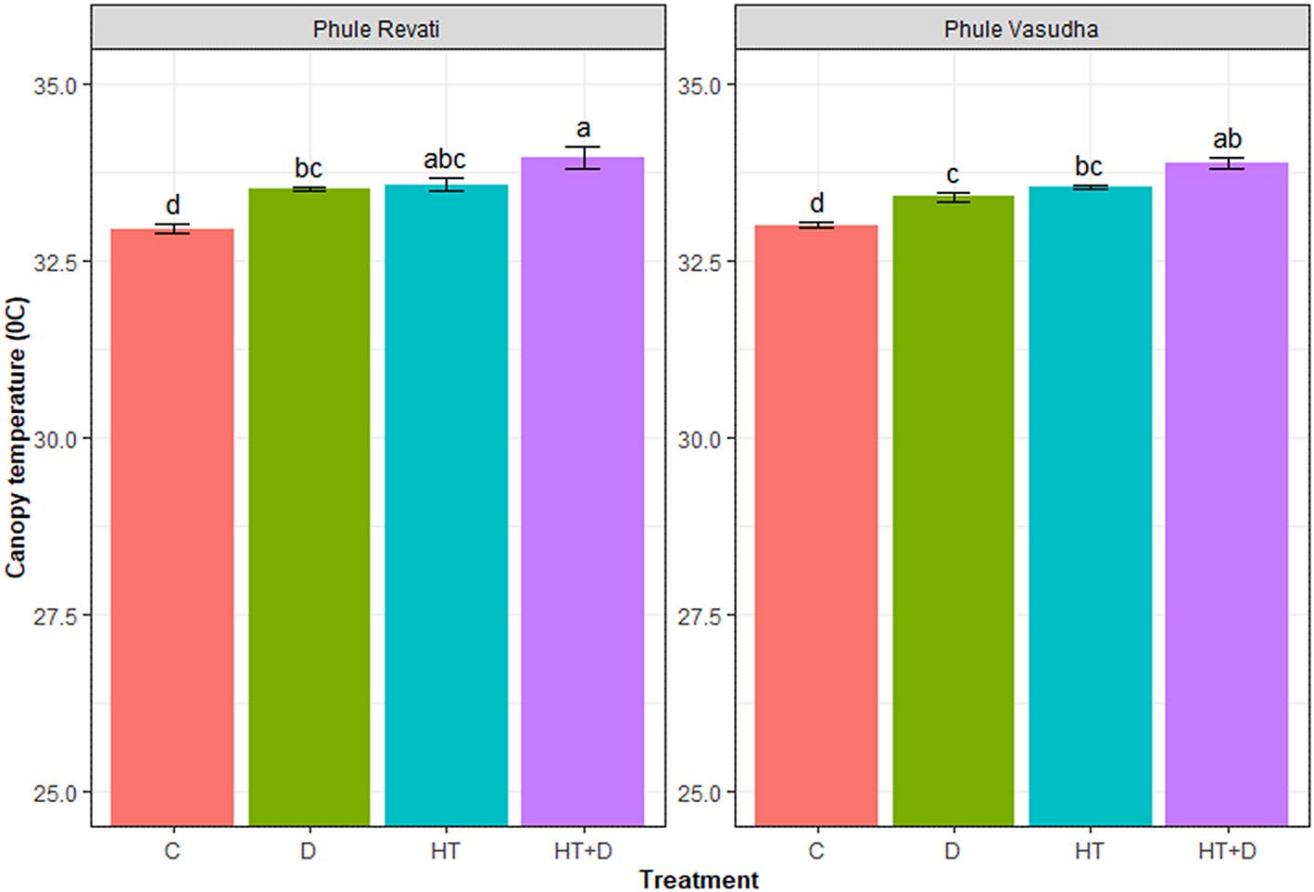
Canopy temperature of Phule Revati and Phule Vasudha under control (C), drought (D), heat (HT), and heat + drought (HT + D) stress treatments. The data represent mean values ± SE (n = 8). Bars with different letters indicate significant differences (P < 0.05).

**Figure 2:**
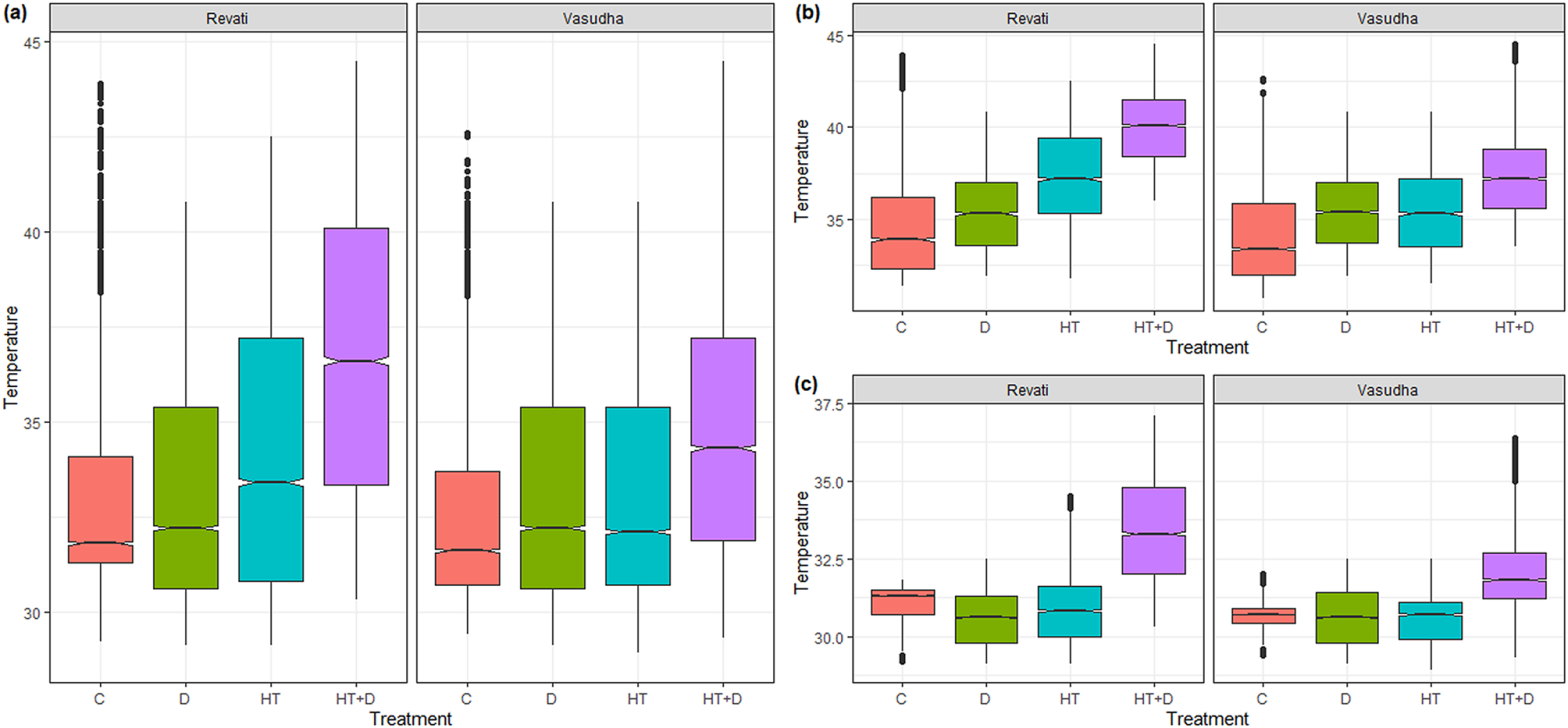
Canopy temperature of Phule Revati and Phule Vasudha under control (C), drought (D), heat (HT), and heat + drought (HT + D) stress at (**a**) analyzed values of all the pixels (**b**) hot pixels and (**c**) cool pixels of the segmented images of plants as explained in material and methods. Each box indicates the interquartile range of canopy temperature under a particular treatment, with the horizontal line representing the median. The notch displays the confidence interval around the median and if the notches of two boxes do not overlap, it indicates the significant difference (P < 0.05).

Analysis of canopy temperature pixels differentiated the cultivars more effectively than the conventional approach of average temperature. As shown in Fig. 1, compared to control, the average canopy temperature was significantly higher under all the stress conditions, irrespective of cultivars. However, there was no clear differentiation among the stress conditions. In contrast, pixel-wise analysis of canopy temperature notably, hot pixels showed a much clear response of cultivars to imposed stress conditions (Fig. 2). The effect of heat on canopy temperature was more conspicuous than that of drought when assessed separately. However, the combined effect of drought and heat on the enhancement of canopy temperature was comparatively higher than any of the stress imposed individually. The significant negative impact of stress conditions was found on the cultivars with Phule Revati following an increasing trend of C < D < HT < HT + D, whereas in Phule Vasudha, it was C < D ~ HT < HT + D (Fig. 2b). However, under similar stress conditions, Phule Vasudha had the ability to keep its canopy relatively cooler as compared to Phule Revati.

### Comparative response of membrane stability index, chlorophyll content, and relative water content to stress treatments

Mean values for physiological traits revealed stress-specific responses of plants. The membrane stability was highly affected by heat stress. Compared with control, high temperature stress independently or in combination with drought reduced membrane stability index to the extent of 25 to 30% (Table 1). The leaf chlorophyll content of sorghum cultivars decreased under stress treatments as compared with control. However, the adverse effects of high temperature were more damaging for chlorophyll than drought stress. Compared with control, total chlorophyll content was reduced by 44% under combined heat and drought stress, 36% under heat, and 20% under drought, respectively. A similar trend was found for chlorophyll a and b. Between the cultivars, the reduction values for chlorophyll content and MSI were more severe for Phule Revati than for Phule Vasudha. Further, drought stress alone reduced the RWC by 25%, and the damage was enhanced (41%) in combination with heat stress. However, both the cultivars maintained a similar level of tissue hydration (Table 1).

**Table 1:** Effect of stress treatment on chlorophyll content, membrane stability index, and relative water content of Phule Revati and Phule Vasudha.

Treatments	Chlorophyll a (mg g^−1^ FW)		Chlorophyll b (mg g^−1^ FW)		Total chlorophyll (mg g^−1^ FW)		Membrane stability index (MSI) (%)		Relative water content (RWC) (%)	
	Phule Revati	Phule Vasudha	Phule Revati	Phule Vasudha	Phule Revati	Phule Vasudha	Phule Revati	Phule Vasudha	Phule Revati	Phule Vasudha
Control (C )	2.17 ± 0.09a	2.16 ± 0.03 a	0.66 ± 0.03 a	0.70 ± 0.03 a	2.83 ± 0.12 a	2.86 ± 0.06 a	50 ± 5 a	55 ± 2 a	82 ± 2 a	85 ± 3 a
Drought (D)	1.49 ± 0.05b	1.88 ± 0.11 a	0.46 ± 0.02 b	0.66 ± 0.04 a	1.95 ± 0.07 b	2.54 ± 0.15 a	41 ± 1 b	51 ± 2a	59 ± 5 b	65 ± 5 b
Heat (HT)	1.03 ± 0.07 c	1.49 ± 0.09 ab	0.41 ± 0.01 c	0.58 ± 0.09 ab	1.44 ± 0.08 c	2.07 ± 0.18 ab	35 ± 2 c	44 ± 2 ab	79 ± 2 a	83 ± 3 a
Heat + drought (HT + D)	0.88 ± 0.04 c	1.30 ± 0.02 b	0.39 ± 0.04 c	0.49 ± 0.02 b	1.37 ± 0.08 c	1.79 ± 0.04 b	31 ± 1 d	40 ± 3 abc	41 ± 6 c	57 ± 7 c

The data represent mean values ± SE (n = 8). Values with different letters indicate a significant differences with P < 0.05.

### Comparative response of chlorophyll fluorescence to stress treatments

**Figure 3:**
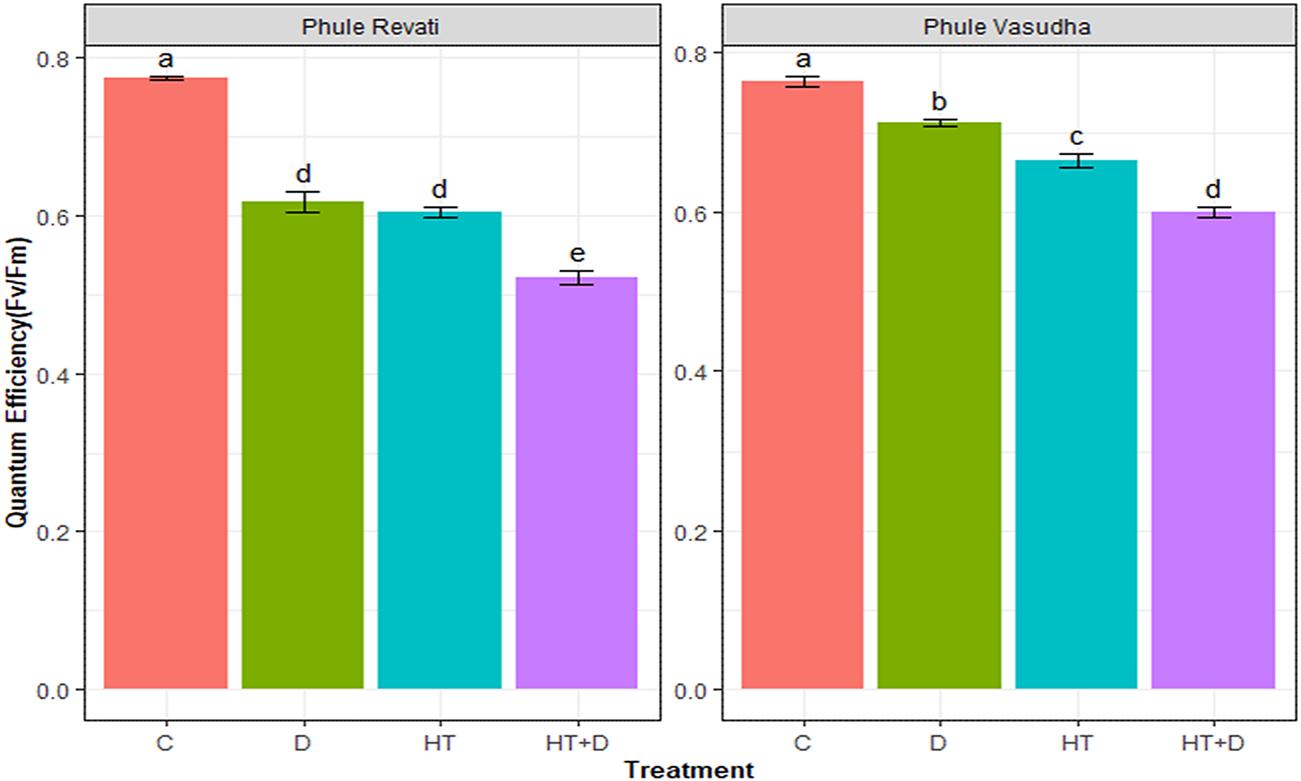
Quantum efficiency of PS II (F_v_/F_m_) of Phule Revati and Phule vasudha under control (C), drought (D), heat (HT), and heat + drought (HT + D) stress at (**a**) 5th day after stress treatment and (**b**) 10th day after stress treatment. The data represent mean values ± SE (n = 8). Different small letters above the bars indicate significant differences (P < 0.05).

Chlorophyll fluorescence measurement indicating the PS-II maximum quantum efficiency (F_v_/F_m_) ranged from 0.78 to 0.50 under the stress treatments (Fig. 3). Drought, heat, and combined stress decreased F_v_/F_m_ by 15, 22, and 38%, respectively, compared with the control and averaged across the cultivars. Under control conditions, the cultivars were not significantly different for F_v_/F_m_, and the average value was around 0.77. Overall, the combined stress of drought and heat decreased F_v_/F_m_ by 33% in Phule Revati (highest decline). In contrast, the lowest reduction (13%) in F_v_/F_m_ was reported under drought stress in Phule Vasudha, as compared to the control. Phule Vasudha showed a clear trend of reduction in PSII quantum efficiency with the highest decline under HT + D (27%) followed by HT (17%), D (13%) as compared to control. However, in Phule Revati, though the highest reduction was seen under HT + D (33%), both drought and heat had a comparable negative impact (20% reduction) compared to control.

### Association between canopy temperature and relative water content with other physiological variables

**Figure 4:**
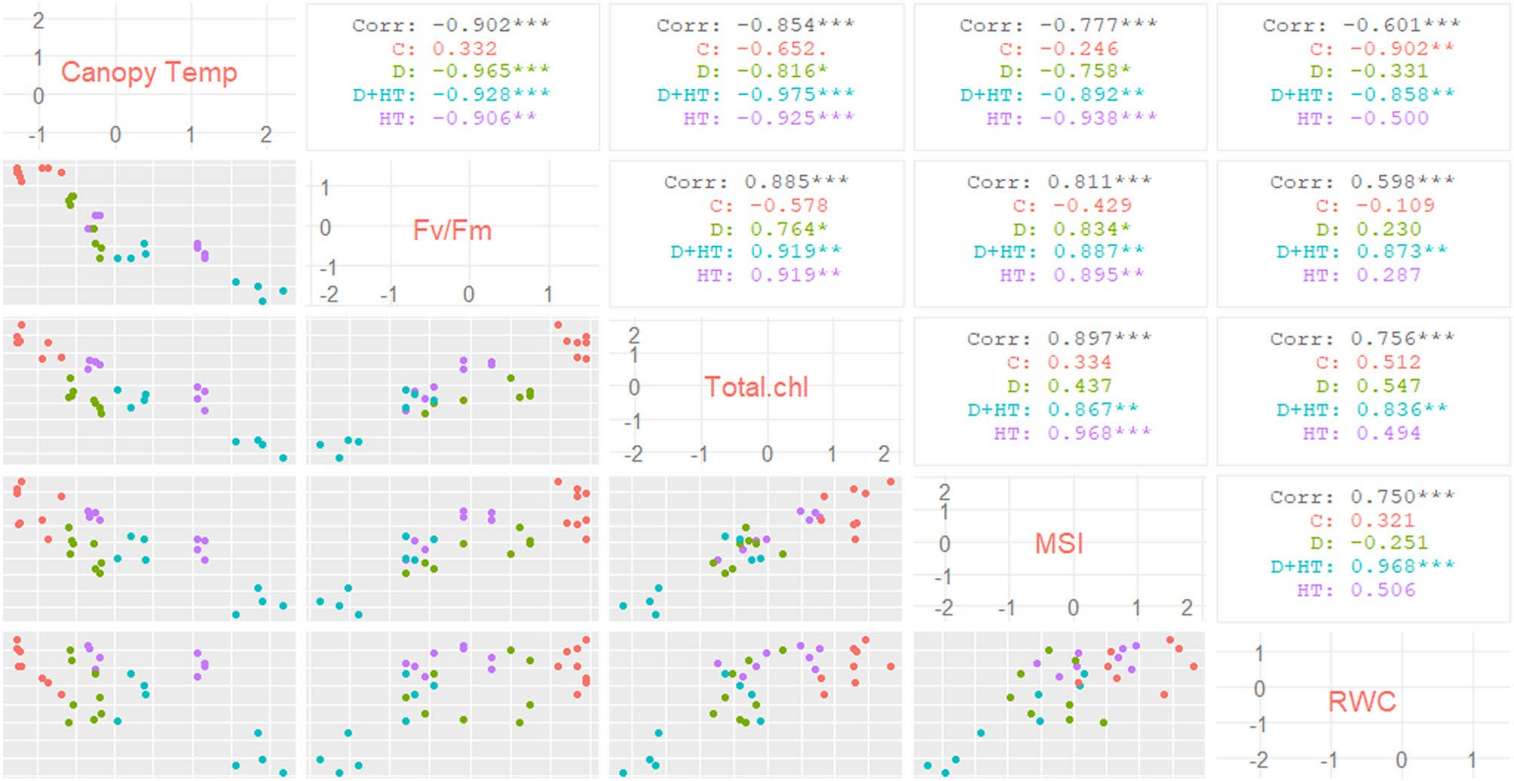
Scatterplots of each pair of traits recorded in the experiment. Pearson correlation is displayed on the right.

**Figure 5:**
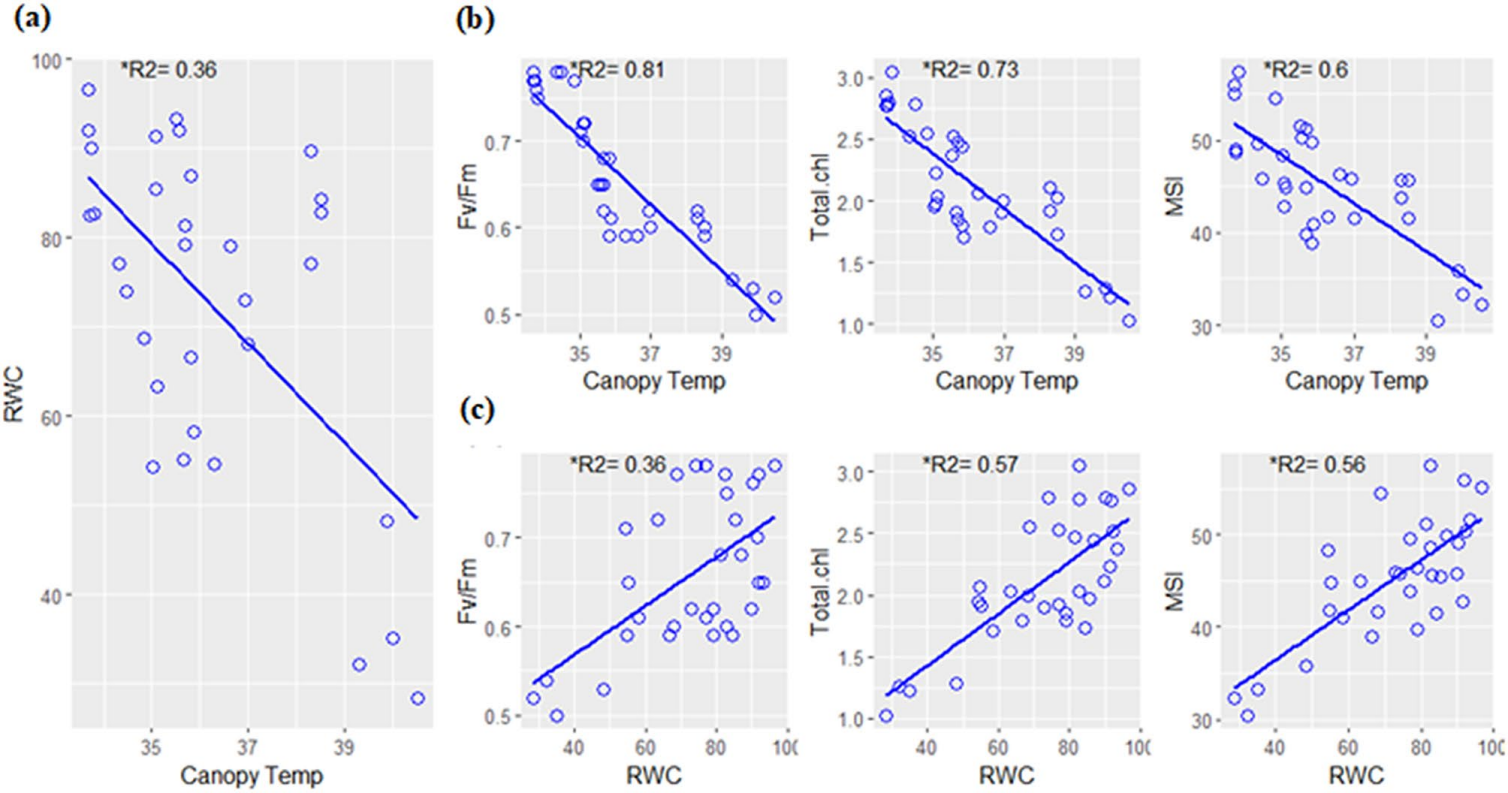
Coefficient of determination (R2) computed to reveal association among variables (A) RWC (%) and Canopy Temperature (°C) (B) F_v_/F_m_, Total Chlorophyll (mg/g fresh weight) and MSI(%) versus Canopy Temperature (°C) and (C) F_v_/F_m_, Total Chlorophyll (mg/g fresh weight) and MSI (%) versus RWC (%).

Pearson correlation coefficient computed for each pair of the traits investigated in this experiment revealed a close association among the traits. However, the variation in critical physiological parameters could be explained more effectively by canopy temperature as depicted by the higher significant correlation values (p < 0.001) with the studied physiological parameters (Fig. 4). In addition, Fig. 5a,b revealed that canopy temperature was negatively related to the studied parameters and could explain 81% variation in quantum efficiency, 73% variation in total chlorophyll content, and 60% of the variation in membrane stability index of the sorghum cultivars whereas variation explained by RWC was substantially less in most cases than that described by canopy temperature (Fig. 5c).

## Discussion

Drought, heat, and a combination of both, applied at seedling establishment stage of sorghum cultivars, was sufficient enough to elucidate differential responses of physiological traits viz., canopy temperature, chlorophyll fluorescence, chlorophyll content, relative water content, and leaf membrane stability at seventh day after treatment imposition. This is in concurrence with earlier findings on grain sorghum that abiotic stress, including drought and heat, affects physiological traits on day seven^3,30^.

The focus of this study was on canopy temperature, as it is one of the crucial parameters suggested to assess the plant responses to stresses imposed by elevated ambient temperature and soil moisture depletion^20^. This can be attributed to the transpiration process regulated by stomatal mechanisms and plants' capacity to extract soil moisture. Previously, infrared guns and, recently, thermal imaging systems have been employed to monitor the canopy temperature of crop plants^21^. In most cases, the average temperature values provided by these instruments are used to assess plants' responses. The average values may not appropriately represent the whole plant canopy temperature as the leaves may get differentially warmed as per their position in the canopy profile and age. We attempted to bridge this gap. Our experiments revealed that estimation of canopy temperature through pixel-wise analysis of thermal images of plants can provide better insight and differentiate both treatment and genotype effects with greater resolution when plants are subjected to stresses caused by heat and drought individually or in combination. This is evident from the linear increase in the canopy temperature represented by the average temperature values of hot pixels in the segmented image of the plant in response to the rise in the level of stress caused individually or in combination (Fig. 3). Hence, the average of hot pixels was considered for further analysis of treatment and genotype effects on critical physiological parameters.

### Response of critical physiological parameters to imposed stress conditions

One of the primary impacts of stresses such as drought and heat on plants is damage to cell membranes crucial for normal cell functions. Hence cell membrane stability has been widely used to assess stress responses of crop plants^12^. As expected, the combined drought and heat stresses decreased membrane stability, leaf chlorophyll and relative water content to a greater extent than by their independent occurrence in both the cultivars. Similar reports of membrane damage caused by drought^31^ and high-temperature stress^30^ have been attributed to the reactive oxygen species (ROS) accumulation, which also triggers degradation of chlorophyll in leaves^32^. Chlorophylls are the primary photosynthetic pigments located in chloroplasts' thylakoid membranes. In general, an appropriate balance between chlorophyll synthesis and degradation enables plants to maintain a nearly constant concentration in plant leaves^33,34^. However, exposure of plants to abiotic stresses, including drought and heat, leads to leaf chlorosis and senescence, primarily owing to its chlorophyll content reduction^35^. In our study, chlorophyll *a*, chlorophyll *b*, and total chlorophyll content was remarkably reduced by both independent and combined stress conditions. Though drought and the high temperature share some common mechanisms, the former stress mainly affects the photosynthetic pigments as well as the membranes of thylakoid^36–38^, while the latter primarily affects the chlorophyll biosynthesis, resulting a reduction in the chlorophyll content^39^. Thus, low chlorophyll content in the plants might be attributed to either reduced rate of chlorophyll biosynthesis or due to its accelerated degradation caused by drought and high temperature. Further, relative water content, an indicator of plant water status, was generally affected by drought and mostly independent of temperature under optimum soil moisture. However, high temperatures exacerbated the impact of drought on RWC when water was withheld, and these results agree with previous reports^40^.

Reduction in photosynthesis efficiency in response to the drought in plants^38^ can be partially attributed to reduced CO_2_ availability after stomatal closure for saving water. This process makes the plant more susceptible to photodamage^41,42^. A decline in maximum quantum efficiency (Fv/Fm) is a good indicator of photoinhibition impairment when plants are exposed to various environmental stresses, including drought and heat^43,44^. High temperature stress damages the photosynthesis process by impairing the photosynthetic pigments^45^ decreasing the photosystem II activity^46–48^ and disturbing the RuBP regeneration capacity^49^. However, combined drought and high temperature stresses adversely affect protein structure and functions, electron transport, and release of ions from the protein-binding sites^15,50,51^.

**Figure 6:**
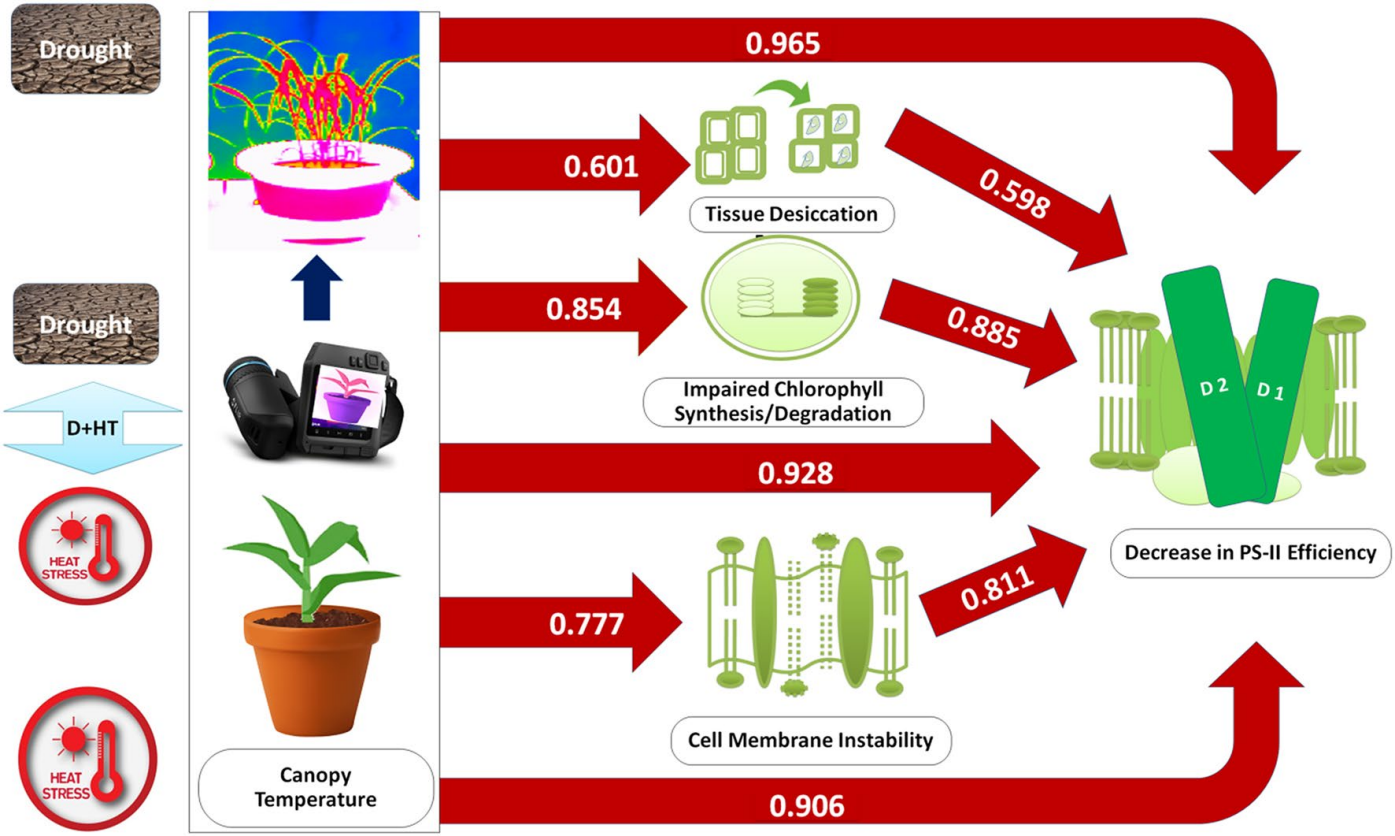
Schematic diagram of the impact of individual and combined effects of drought and heat stress on canopy temperature and other critical physiological parameters in sorghum. Values represent the pearson correlation coefficient between the respective pair of traits (as depicted in Fig. 4). In this figure, the studied parameters viz*.,* RWC, total chlorophyll, MSI and F_v_/F_m_ are represented as physiological processes i.e. tissue desiccation, impaired chlorophyll synthesis/degradation, cell membrane instability and decrease in PS-II efficiency, respectively.

A substantial decrease in PS-II efficiency revealed by the stress-induced changes in Fv/Fm in our experiment could be primarily attributed to a rise in leaf temperature resulted from drought and high temperature stress (Fig. 6). Our study confirms earlier observations that damage to the cell membrane and a decrease in chlorophyll content could be the primary causes of the decline in PS-II efficiency. The variations in these parameters could be explained by variation in canopy temperatures (Fig. 2a,b) with greater certainty. Relatively, the weaker correlation between RWC and other parameters hints that plants like sorghum maintain their tissue water content at a level essential for normal functioning even when exposed to soil moisture stress. However, combined stress due to the drought and the heat led to reduced tissue water content beyond the threshold, nearly 50% of the optimal tissue water content that caused extensive damage to the PS-II system.

### Cultivar effects on canopy temperature and other drought and heat stress induced physiological responses

The primary response of majority of the plants to heat and drought stress is stomatal closure to avoid moisture loss through transpiration. Exposure to drought and heat stress causes a significant decrease in the leaf water potential as well as the transpiration rate, which ultimately increases the leaf and canopy temperature^52^. However, the genotypes that can get adapted to drought stress by conserving the soil moisture at the initial growth phases through optimal transpiration and then use it effectively for keeping the canopy cool until the grain filling stage^53^. We applied the improved approach involving the average of temperatures of hot pixels in the canopy instead of an average of pixels of the whole plant for effective detection of stress-induced changes in sorghum cultivars. Among the sorghum cultivars, Phule Vasudha had a more remarkable ability to keep its canopy cool than Phule Revati under the imposed stress treatments. Similar findings on differential response of genotypes within a specific plant species to a combination of drought and heat stress are reported^20,54^. Phule Vasudha, being a drought-tolerant variety, might have the ability to balance between conserving moisture and protecting from overheating.

In contrast, Phule Revati might have lower stomatal conductance, owing to higher canopy temperature and hotter plant canopy. Water relations in both cultivars adjusted to high temperature when the soil moisture level at field capacity was maintained, whereas elevated temperature interacted strongly with moisture deficit condition and exacerbated its impact when water was withheld^55,56^. The more severe impact of combined drought and heat stress on leaf temperature than their individual impacts are in agreement with^24,25,51^. Similarly, under combined stress, reduction in quantum efficiency was higher than that of independent drought or high-temperature stress events in both the cultivars, but the level of damage was lesser in Phule Vasudha than in Phule Revati. The reduced values of Fv/Fm in the leaves of Phule Revati indicated that both drought and heat, independently and in combination, caused a great extent of photoinhibition in photosystem II units of this cultivar in contrast to Phule Vasudha. These interpretations derive support from previous reports about reducing photosynthetic efficiency under drought and heat stress when imposed either individually or in combination^14,51,57–59^.

Drought tolerant crops like sorghum prioritize the establishment of their root system during depletion of soil moisture coupled with high temperature which is common in semiarid regions. Early establishment can enable the plants to explore the soil moisture from deeper layers of soil profile. This can be indirectly reflected by canopy temperature of plants since transpiration is the major cause of changes in leaf water status and leaf temperature^60^. Since maintenance of a cool canopy is a promising trait for identifying drought tolerance^10^, there is high certainty that the seedling stage screening reported in this study will have implications for other critical stages of growth.

## Conclusion

This study revealed that estimation of canopy temperature based on hotter pixels of thermal images of plants can differentiate the stress responses of sorghum cultivars more effectively than the average temperature. Comparison of two cultivars of sorghum differing in maintaining their canopy coolness indicated that primarily the canopy temperature and then the tissue hydration level reduced the photosynthetic efficiency of plants by impairing cell membrane stability and reducing chlorophyll content when exposed to stresses. Hence, the combined effect of drought and the high temperature was more prominent than their individual effects as the continued loss of water coupled with high-temperature exposure exacerbated the adverse impact of stresses with a remarkable increase in canopy temperature and reduction in the tissue water content to nearly 50% of the optimal level. The improved method that emerged from this study to differentiate the sorghum genotypes under combined stress of drought and high temperature can be helpful in the selection of promising genotypes among the breeding lines and in integrating the concept in the precision water management in crops like sorghum.

## Materials and methods

### Plant materials

Two sorghum cultivars, Phule Vasudha (RSV/423/SPV 1704) and Phule Revati (RSV 1006/SPV 1830), popularly grown under rainfed conditions in arid and semi-arid regions of India, were used in this study. While Phule Vasudha is a drought-tolerant variety, no such information is available on abiotic stress tolerance for Phule Revati. Quality seeds were obtained from the sorghum improvement project at MPKV, Rahuri, India.

### Experimental setup and growth conditions

This research was conducted at the control environmental facility of the ICAR-National Institute of Abiotic Stress Management (NIASM), Baramati, India (18° 09′ 30.62" N latitude; 74° 30′ 03.08" E longitude; MSL 570 m altitude) in a completely randomized design. Plants were grown in plastic pots (diameter 30 cm and height 42 cm) filled with 14 kg clay loam soil (72% clay, 24.4% and 4% silt) having pH 8.4, EC 0.24 dSm^-1^, organic carbon 6.3 g kg^-1^, 170 kg nitrogen, 17 kg phosphorous, and 140 kg potash ha^-1^. Ten seeds per pot were sown at a depth of 3–4 cm. A week after emergence, they were thinned to six seedlings per pot and maintained at normal conditions until they were shifted to the greenhouse for stress treatments 15 days after emergence. The plants were exposed to drought stress [D] by shifting in the greenhouse with regular day/night (35/20 °C) temperature, and maintaining the moisture content at 15% field capacity. Heat stress [HT] was imposed by shifting plants to the greenhouse with 40/25 °C day/night temperature and regular irrigation (i.e., 50% of field capacity) while a set of pots were kept with soil moisture level at 15% field capacity to impose a combination of heat and drought stress [HT + D] in the same greenhouse. The non-stressed plants [C] were maintained with regular irrigation and 35/20 °C (day/night) temperature. The water holding capacity of the pot containing 14 kg of soil was predetermined following a gravimetric method^61^. The stress period was for a week, and each treatment had eight replicated pots. Every alternate day, plants in each greenhouse were randomly shifted to avoid any positional effect within the chamber. In order to impose the drought stress resulting from soil (potting mix) moisture deficit, the greenhouses were set at high relative humidity (RH) to avoid the stress occurring from rapid evapotranspiration under elevated air temperatures. Both the greenhouses were maintained at 65–70% RH and 450–750 µmol m^-2^ s^-2^ PAR. After stress treatment, all pots were kept in one greenhouse and were held at a day/night temperature of 35/20 °C and regular irrigation. The pots were kept weed-free throughout the experiment, and no disease or pest incidences were reported; hence no such measures were taken.

### Data collection

There were eight pots per treatment per cultivar in each replication, and each pot had six seedlings. Fully emerged third leaf at the top of randomly selected seedlings of each pot were chosen for measuring canopy temperature, membrane stability index, chlorophyll content, relative water content, and chlorophyll fluorescence. All data were collected on an alternate day, from the start of the experiment for seven days and all the methods were performed in accordance with the relevant guidelines and regulations.

#### Canopy temperature

**Figure 7:**
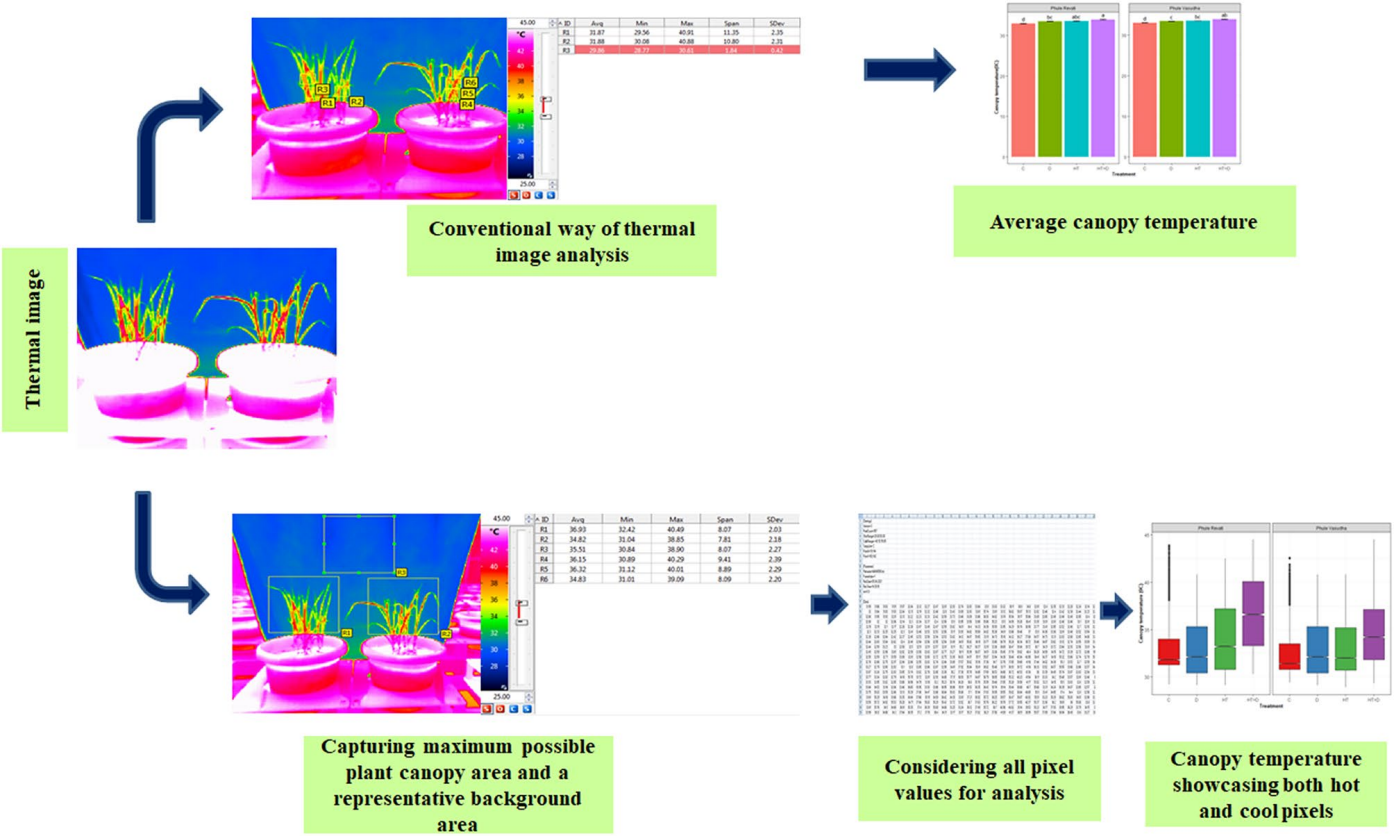
Conventional versus suggested way of analyzing thermal images of canopy temperature.

Thermal images with spatial resolution of 768 × 576 pixels were acquired between 13 and 14 h for measuring canopy temperature of plants under each treatment with a thermal imager (Vario CAM hr. inspect 575, Jenoptic, Germany) of 0.01 °C thermal resolution that operates in the wavelength range of 8–14 μm. A wet cloth was used to ensure the contrasting background to enable segmentation of plant canopy during the image analysis. The ambient temperatures during the measurements were nearly constant at 35 °C in C and D while it was 40 °C in HT and HT + D treatments respectively. The captured radiometric images were saved and within each thermal image, the areas of interest for analysis was selected to account plant canopy area to the extent possible using “IRBIS 3.1 professional” software (Variocam, Jenoptic, Germany). Provision in the software to segment plant from the background was used and temperature revealed by every pixel in the segmented image was considered for data analysis and analyzed using R programme. Deviating a bit from conventional way of measurements of average canopy temperature of the cultivars under each treatment, we considered temperature values of every pixel in the segmented image to interpret response of canopy temperature as well as its influence on physiology of plants (Fig. 7). An average of 50% of the pixels either hot or cool with median value as center of measure were computed separately in the each of the segmented images.

#### Leaf membrane stability index

The membrane stability index (MSI) was estimated by harvesting one fully expanded third leaf at the top of four plants under each replication per cultivar. Collected leaves of all the treatments were cut into small discs (1 cm^2^ size) and 0.5 g of it was placed in test tubes having10 mL of double distilled water and set in duplicates. One set was maintained at 40 °C for 30 min, and another set was kept in a hot water bath of 100 °C for 15 min. Their electrical conductivity (C1 and C2, respectively) were measured by a conductivity meter (Adawa-260, Thermo Scientific, USA), and calculation of MSI was done using the formula, MSI (%) = 1 − C1/C2 × 100^62^.

#### Chlorophyll content

Fully expanded third leaves at the top of each plant from all treatments were collected, and the DMSO method was followed for estimating the chlorophyll content. Leaf samples (25 mg each) were cut into fine strips/ pieces and kept in a test tube containing 5 mL of DMSO. Afterward, the test tubes were incubated in the dark at 37 °C for 24 h for chlorophyll extraction. An aliquot of 3 mL was used for spectrophotometric (UV-1800, Shimadzu, Japan) measurement at 649 and 665 nm to determine chlorophyll a, chlorophyll b, and total chlorophyll contents^63^.

#### Relative water content

Fully expanded third leaf at the top of each plant from all treatments was cut, and the fresh weight (FW) was measured immediately. Then, leaves were kept in distilled water under average room temperature for four hours, and their turgid weight (TW) was measured. Afterward, the same set of leaves were put in a drying oven at 60 °C for 48 h and weighed to obtain dry weight (DW). Finally, the relative water content (RWC) of the leaves was estimated using the equation, RWC (%) = (FW − DW)/ (TW – DW) × 100^64^.

#### Chlorophyll fluorescence measurement

Chlorophyll fluorescence analysis investigates plant photosynthetic performance as it indicates the status of PSII that drives photosynthesis^65^. A sampling of fully expanded third leaves at the top was carried out at around 09:00 h from each replication, and samples were then transferred to a dark room and were adapted and stabilized for the next one h under dark. Dark-acclimatized and stabilized samples were collected, and chlorophyll fluorescence was recorded on each sample with the help of an imaging fluorometer instrument (Handy FluorCam, P.S.I., Brno, Czech Republic) as reported in^66^. Fluroscence was detected by a high-sensitivity charge coupled device camera and it was driven by *FluroCam* software package (*FluorCam* 7 version 1.2.5.3). First, non-actinic measuring flashes provided by super-bright light-emitting diodes (LEDs) were used to obtain the dark-adapted fluorescence level (F_0_) images. Next, the maximum fluorescence level (F_m_) was measured through an 800-ms duration pulse of saturating light radiation [2,500 µmol (photon) m^-2^ s^-1^] generated by a halogen lamp. Indicator of chlorophyll fluorescence, i.e., maximal photochemical efficiency of PSII (F_v_/F_m_), was calculated as (F_m_ – F_0_)/F_m_ where variable fluorescence (F_v_) represents the difference between F_m_ and F_0_.

### Statistical analysis

Statistical analyses were executed with general linear models (GLMs) using the “Agricolae” package of R (R Development Team, 2011). The experimental setup was a completely randomized design. Plants were selected randomly for treatments as well as kept randomly within the greenhouses. The physiological parameters were measured in randomly selected plants from each replication under each treatment. For all the data, an estimate of variability was shown by the standard error, and significance of variable means was performed by the Least Significant Difference (LSD) test at 5% probability level. Wilcoxon test was performed for showcasing the differential response of canopy temperature to imposed stress conditions^67^. Besides, correlation analysis was done to relate the canopy temperature, and relative water content and their relationship with membrane stability index, total chlorophyll content and chlorophyll fluorescence.

## Data Availability

All the relevant data supporting the conclusions of this study are included within the article.
